# Investigation on the Effect of Different Mild Acidic Electrolyte on ZIBs Electrode/Electrolyte Interface and the Performance Improvements With the Optimized Cathode

**DOI:** 10.3389/fchem.2020.00827

**Published:** 2020-10-23

**Authors:** Yang Gui, Yang Lei, Bao An Fan

**Affiliations:** Key Laboratory of Hubei Province for Coal Conversion and New Carbon Materials, School of Chemistry and Chemical Engineering, Wuhan University of Science and Technology, Wuhan, China

**Keywords:** aqueous electrolye, interfacial effect, ZIB performance, electrochemical assessment, Zn stripping/plating kinetics

## Abstract

Zinc ion batteries (ZIBs), as promising alternatives of lithium ion batteries (LIBs), have aroused revived interest in the energy storage field recently. To obtain good performance, the choice of electrolyte plays a vital role. Therefore, in this work, three kinds of aqueous electrolyte have been assessed for interfacial effect and ZIB performance. Through the comparison, ZnSO_4_ electrolyte showed advantages on ionic conductivity over both Zn(NO_3_)_2_ and Zn(CH_3_COO)_2_ solution, Zn(CH_3_COO)_2_ exhibited considerable Zn stripping/plating kinetics with ZnSO_4_, and there was no characteristic peak pair of Zn dissolution/deposition found in Zn(NO_3_)_2_. Additionally, a lower charge transfer resistance was proved when the cell with 1 M ZnSO_4_ solution. Besides, to further study on cathodic graphite paper, activation and optimization has been conducted, and cells with optimized graphite paper showed an outstanding enhancement.

## Introduction

Nowadays, different types of rechargeable batteries, as lithium-ion battery (LIB) alternatives, have attracted attention of researchers due to that (1) limited sources of lithium; (2) higher flammable risk of organic electrolyte in LIBs; (3) high chemical activity of lithium leading to a harsh requirement on its preparation process (Whittingham, 2004; Armand and Tarascon, 2008). Therefore, it is significant to search for new ion battery resources with considerable or even higher capacity but much safer and convenient fabrication methods. Systems built up on elements with electropositive characters such as Na, Mg, Al, Ca, and Zn have attracted extensive interest because of promising theoretical capacity, abundant material and effective cost (Li et al., 2014; Muldoon et al., 2014; Yabuuchi et al., 2014; Lin et al., 2015; Choi and Aurbach, 2016; Ponrouch et al., 2016; Zhang et al., 2016). Meanwhile, replacing the organic electrolyte with an aqueous one offers advantages including more convenient manufacturing conditions as well as advantageous cost, greener, safer, and better ionic conductivity (Lu et al., 2011; Kim et al., 2014). Regarding these superior features, aqueous Zn-ion battery (ZIB)/capacitor (ZIC) technology plays a particular role as a promising alternative based on its property of good water compatibility, low flammability and, especially when compared with LIBs, Zn exhibits higher charge density (Lee et al., 2015; Pan et al., 2016).

Following the development history of ZIBs, the first ZIB prototype was made in 1999 by using zinc metal as electrode and alkaline as electrolyte which presented excellent characters of low redox potential [−0.76 V vs. standard hydrogen electrode (SHE)]. Since then, Zinc metal has been applied widely in different batteries like Ni-Zn battery, MnO_2_-Zn battery, Zinc ion battery, and Zinc air battery (Yang and Lin, 2002; Cheng et al., 2005). However, the sever zinc dendrite formation in alkaline solution extremely shortened its cycling life and limited the corresponding discharge capacity. To deal with this problem, the displacement of alkaline electrolyte with mild solution of zinc sulfate was firstly investigated by Yamamoto and Shoji (1986). According to the observation, due to mild acidic condition, the appearance of ZnO, Zn(OH)_2_, etc. by-products on zinc anode has been effectively inhibited (Yamamoto and Shoji, 1986). Hence, the choice of electrolyte plays a vital role for the overall performance of ZIBs. Firstly, the function of the electrolyte does not only provide pathways for ion migration but also determines the reversibility of Zn plating/stripping process and SEI formation (Wang F. et al., 2018; Wang Z. et al., 2018; Qiu et al., 2019; Yuan et al., 2019; Zhao et al., 2019). Secondly, the accurate choice of electrolyte, including its concentration, pH value, coordination number and so on, could maximize the energy storage capacity of the whole system to achieve its optimized performance (Li et al., 2016; Wang F. et al., 2018; Wang Z. et al., 2018; Zhao et al., 2018). Up to now, the exploration on electrolytes for ZIBs is still in its infancy, and more details on the understanding of the effect of electrolytes on the electrochemical performance is required. In this paper, to reveal the relationship between electrolyte and zinc storage performance, we investigated three kinds of electrolyte by using zinc and graphite paper as counter and working electrode, respectively, from the perspectives of stripping/plating kinetics, interfacial kinetics, and its behavior on zinc storage performance. Furthermore, cathode optimization has been designed as well and harvested outstanding enhancement on ZIB performance.

## Experiments

### Chemicals and Materials

Zinc acetate dihydrate (ALADDIN); Zinc nitrate hexahydrate (ALADDIN); Zinc sulfate monohydrate (ALADDIN); Ethanol (ALADDIN); concentrated sulfuric acid (ALADDIN); Acetone (ALADDIN). Zn plate (34 × 150 × 0.2 mm); Graphite paper (125 × 200 × 0.1 mm).

### Material Pretreatment

Zn plate was cut into a dimension of 34 × 25 × 0.2 mm, then placed into a mixture solution of ethanol and acetone with a volume ratio of 1:1, followed by exposure to an ultrasound bath for 15 min, after that the sample was taken out and readied for later use. The graphite paper wascut into a dimension of 30 × 20 × 0.1 mm and cleaned with acetone.

The activation of graphite paper was realized by exposing one clean piece of graphite paper in a three-electrode system at a constant potential for a certain period. The potential sweep rate was set at 0.01 V s^−1^. The system was constructed by a piece of graphite paper, platinum foil, and saturated calomel (SCE) as working, counter, and reference electrode, respectively. The electrolyte was composed by 2 M H_2_SO_4_. Five samples were prepared and named as S1, S2, S3, S4, and S5, respectively. S1 was treated by exposing one piece of graphite paper in 2 M H_2_SO_4_ under 4 V for 60 s. When the treatment was finished, S1 was taken out and flushed by DI water until pH of the solution close to ~6.7 (pH value of the DI water), then placed in an oven at 60° overnight. The treatment condition for S2 was the same as S1 except the final potential at 6 V for a period of 300 s. S3 and S4 was treated at 6 V as well but within a period of 450 and 600 s independently. S5 was activated at 8 V for 450 s.

### Electrochemical Tests

#### Three Electrode System Measurement

The testing system was constructed by a piece of graphite paper with a dimension of 2 × 3 cm^2^ as the working electrode, a piece of Zn plate (2.5 × 3.5 cm^2^) as the counter electrode and another piece of Zn plate (2.5 × 3.5 cm^2^) as the reference electrode. The series of cyclic voltammograms (CVs) in different electrolytes were recorded at a potential sweep rate of 1 mV s^−1^ within a proper electrochemical window. Electrochemical impedance spectroscopy (EIS) measurement was conducted through a CHI600E electrochemical workstate with an AC voltage of 5 mV amplitude in the frequency ranging from 100 kHz to 100 mHz. This measurement was used to differentiate the effect on different electrolyte on electrode interfaces when considering a ZIB performance.

#### Two Electrode System Measurement

The two-electrode system consisted of using a CR2032 coin-type. CR2032 coin cell was assembled by the pristine or activated graphite paper as a working electrode, Zn foil as a counter electrode, filter paper as a membrane and 1 M ZnSO_4_ as an electrolyte. The galvanostatic charge/discharge performance of the cell has been conducted by a LAND-CT2001A battery-testing instrument.

### Morphology Characterization

Scanning electron microscopy (SEM) was taken by Nova NanoSEM400 microscopes equipped with energy dispersive spectroscopy (EDS) for elemental analysis.

## Results and Discussion

**Figure 1:**
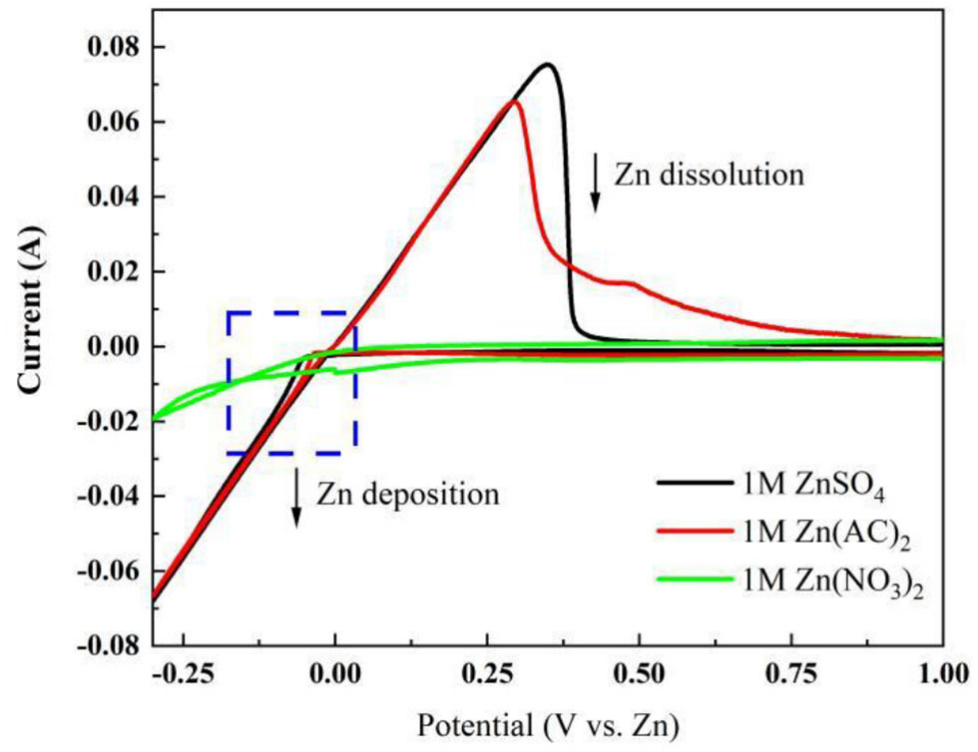
Cyclic voltammetry characterization in a three-electrode system at a scan rate of 1 mV s^−1^ in different electrolytes.

A three-electrode system consisting of Zn/Zn/graphite paper as counter/reference/working electrode wasbuilt up. Three kinds of electrolytes havebeen selected. As shown in Figure 1, the cyclic voltammetry in Zn(CH_3_COO)_2_ and ZnSO_4_ electrolyte reveals an obvious reversible electrochemical stripping/plating process of Zn (Xu et al., 2012; Li et al., 2016). However, no characteristic peak pair corresponding to Zn dissolution/deposition has been found in 1 M Zn(NO_3_)_2_ due to the instability of ${\text{NO}}_{3}^{-}$ (Zhang et al., 2016). Notably, the onset potential for deposition/dissolution of Zn plating/stripping is −0.13/−0.034 V and −0.11/−0.055 corresponding to Zn(CH_3_COO)_2_ and ZnSO_4_ separately (Liu et al., 2016, 2017). Compared with ZnSO_4_, a smaller potential separation was observed in Zn(CH_3_COO)_2_ which reflects better reversibility. Furthermore, a lower oxidation peak at around 0.48 V is suspected due to the oxidation of CH_3_COO^−^. However, the higher current density exhibited in ZnSO_4_ solution assists a faster kinetics. Therefore, from the cyclic voltammetry analysis on these three electrolytes, it is concluded that (1) there is no obvious Zn stripping/plating process captured in 1 M Zn(NO_3_)_2_ electrolyte at the corresponding electrochemical reactive potential range; (2) relative to 1 M Zn(CH_3_COO)_2_ solution, the system in 1 M ZnSO_4_ behaves faster kinetics of Zn dissolution/deposition.

**Figure 2:**
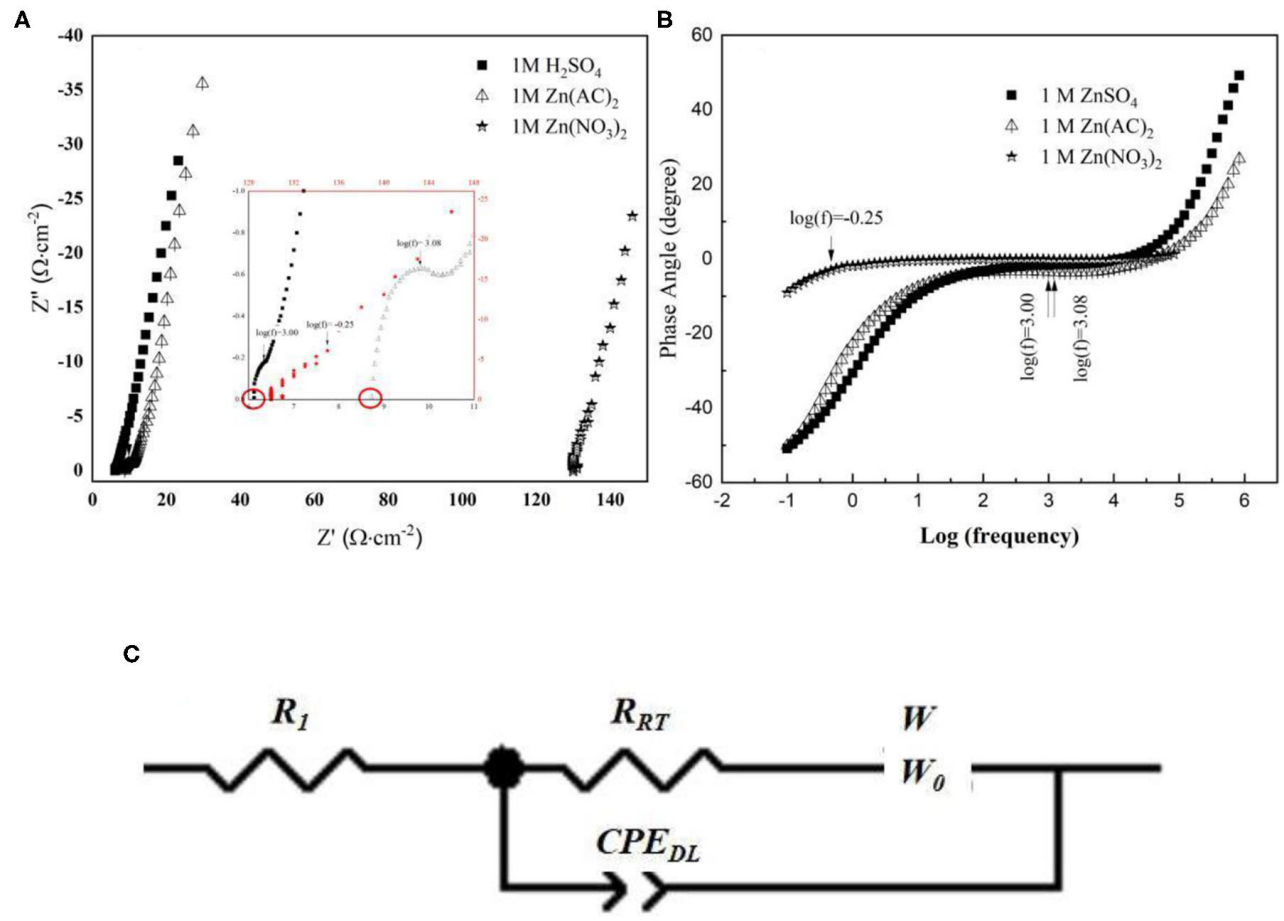
**(A)** EIS Nyquist plots and Bode plots **(B)** collected from a three-electrode system with different electrolyte; **(C)** Equivalent Circuit for data fitting.

To further investigate the interface effects between the electrolyte and electrode surface, an electrochemical impedance spectrum (EIS) was designed. The measurement was set at open circuit potential with respect to the chosen three electrolytes as shown in Figure 2. In their Nyquist plots in Figure 2A, through the inset image, a depressed semicircle in the high-frequency region can be seen in all these three electrolytes. In further detail, the cross section at the X-axis tells resistance difference due to the different ionic conductivity of respective electrolyte (Hwang, 2018). Hence, the result may indicate an ionic conductive sequence of 1 M ZnSO_4_, 1 M Zn(CH_3_COO)_2_ and 1 M Zn(NO_3_)_2_ from high to low. In addition, the highest imaginary point of the enlarged semicircle in the high-frequency region corresponds to the phase transition in their Bode plots in Figure 2B (Wang et al., 2001), which represents a charge transfer process involving stripping/plating of Zn/Zn^2+^. Based on their characteristic frequencies, the lowest one corresponds to 1 M Zn(NO_3_)_2_ and indicates a slower kinetics while the other two show higher kinetics, which is in agreement with their cyclic voltammetry observation. Furthermore, the platform extended farthest to 60° describes a diffusion process, which is characterized by the size of warburg. The size of warburg time constant is inversely proportional to the square-root of Zn^2+^ diffusivity (Gui and Blackwood, 2015). The fitting results based on the equivalent circuit displayed in Figure 2C were listed in Table 1, where *R*_*s*_ represents the series resistance originated from electrode, electrolyte, and the contact; *CPE*_*DL*_ denotes a constant phase element designating the double layer capacitance arising from the charges accumulated on the surface of electrode; *R*_*CT*_ is the resistance produced by the charge transfer process from the electrolyte to Zn surface, which also associates with this process kinetics; and *Wo* is symbolized as an open circuit Warburg impedance reflecting ion diffusion from electrolyte to the electrode/electrolyte interface. From Table 1, the series resistance in 1 M ZnSO_4_ is the lowest and the highest one is assigned to 1 M Zn(NO_3_)_2_, which is suspected to be caused by the different solvation condition in different electrolytes and further the various ionic conductivity. From this aspect, it means that ZnSO_4_ solution should be the most preferred electrolyte with favorable ionic conductivity, hence benefit for ions diffusion. Simultaneously, the value of *R*_*CT*_ also keeps the same sequence with *Rs*. The smaller charge transfer resistance in ZnSO_4_ demonstrates a faster kinetics which is consistent with the anodic current density recorded in Figure 1. Additionally, a highest value of *CPE*_*DL*_ in 1 M ZnSO_4_ electrolyte was captured as well which could be due to the faster charge transfer kinetics making a rougher surface of the Zn electrode. However, the system with 1 M ZnSO_4_ exhibits the longest time period for ions diffusion onto electrode surface that may indicate a longer path length the ions in 1 M ZnSO_4_ will travel. This elongation may be due to the formation of Zn(OH)_x_(SO_4_)_y_·zH_2_O on the electrode surface which takes up the near surface for ions to reach and a further study on SEI on Zn has to be designed to clear the observation (Lee et al., 2016; Pan et al., 2016; Parker et al., 2017).

**Table 1:** Fitting results list based on the equivalent circuit in Figure 2C.

**System with different electrolyte**	***R_***s***_* (Ω cm^**−2**^)**	***R_***CT***_* (Ω cm^**−2**^)**	***W***		***CPE_DL_* (F cm^−2^)**	
			***W-P***	***W-T***	***CPE-T***	***CPE-P***
1 M ZnSO_4_	6.15	0.55	0.61	17.61	0.0023	0.75
1 M Zn(CH_3_COO)_2_	8.82	0.82	0.35	0.12	9.90 ×10^−6^	1.00
1 M Zn(NO_3_)_2_	126.44	3.03	0.61	11.72	3.15 × 10^−6^	0.65

**Figure 3:**
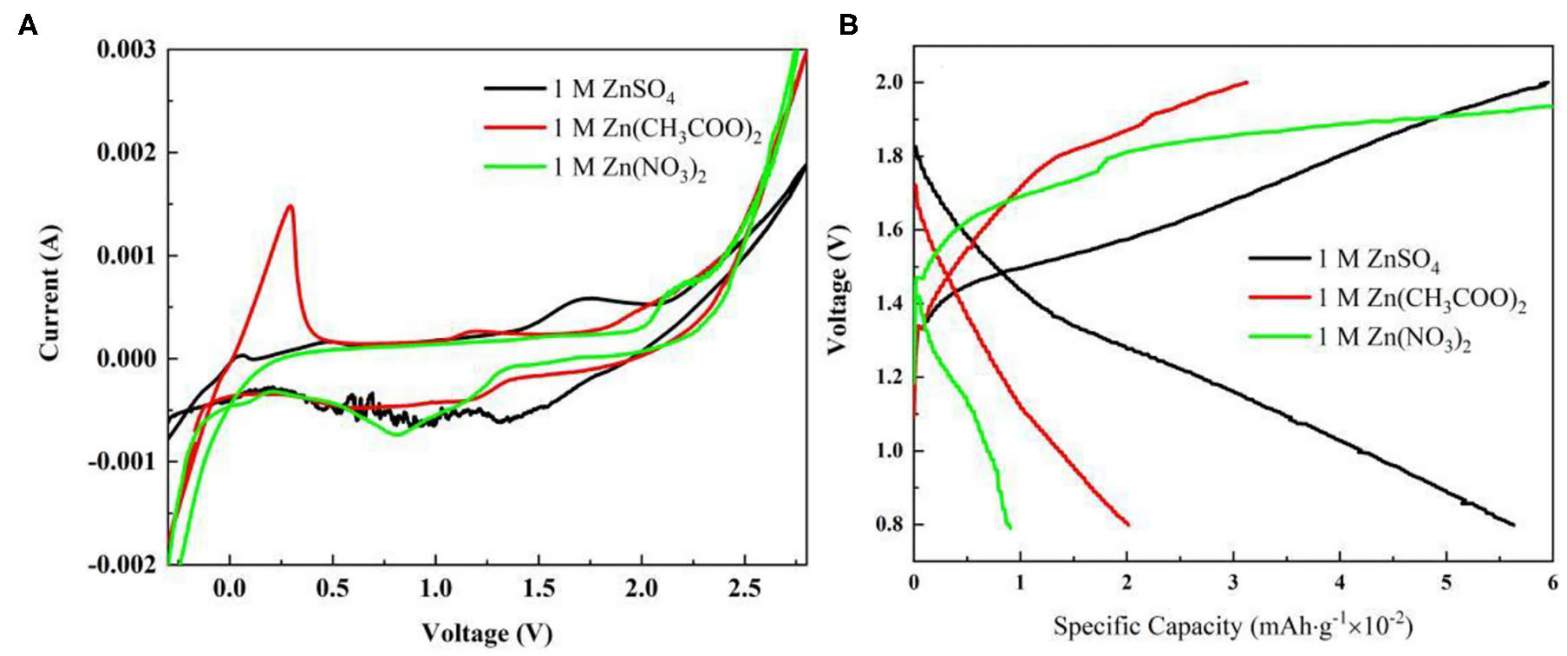
**(A)** Electrode Electrochemical performance characterization in different electrolyte using coin cell construction: **(A)** Cyclic voltammetry (CV) of coin cells at a scan rate of 1 mV s^−1^; **(B)** Galvanostatic charge/discharge curves of coin cells at a current density of 0.02 A g^−1^.

To explore the impact of different electrolytes on ZIB performance, CVs of the coin cell with different electrolytes has been performed. The coin cell was constructed by Zn foil and non-activated graphite paper as a counter and working electrode separately. From Figure 3A, shapes of CVs for both 1 M Zn(CH_3_COO)_2_ and 1 M Zn(NO_3_)_2_ are almost the same as in a three-electrode system, but a sharp decrease on the deposition/dissolution peak pair was observed for a coin cell with 1 M ZnSO_4_ electrolyte, which could be due to formation of a surface layer (Konarov et al., 2018). Additionally, the cyclic shapes enclosed from 0.8 to 2.0 V for the three electrolytes are all close to rectangular, which can be contributed to by the capacitive property of the graphite paper. Furthermore, the ratio of anodic area over its correlated cathodic one is closer to 1 in 1 M ZnSO_4_. This good symmetric behavior implies a better reversibility. In order to reveal the impact of these chosen electrolytes on ZIBs performance, the respective charge/discharge polarization curves at a current density of 0.02 A g^−1^ has been collected and shown in Figure 3B. The overall specific capacities of the ZIBs are low due to the low surface area of the working electrodes, i.e., the graphite papers. Even though, under the same condition, the different behaviors due to various electrolytes are still distinguishable. Figure 3B points out that the ZIB with 1 M ZnSO_4_ performs the highest potential on the electrochemical energy storage capacity. Besides, the good symmetry of its charge/discharge polarization curve reflects the high coulomb efficiency, which is consistent with its symmetric cyclic voltammetry curve. However, coulomb efficiencies of the ZIBs with 1 M Zn(NO_3_)_2_ and Zn(CH_3_COO)_2_ are only 47.4 and 64.8% individually.

**Figure 4:**
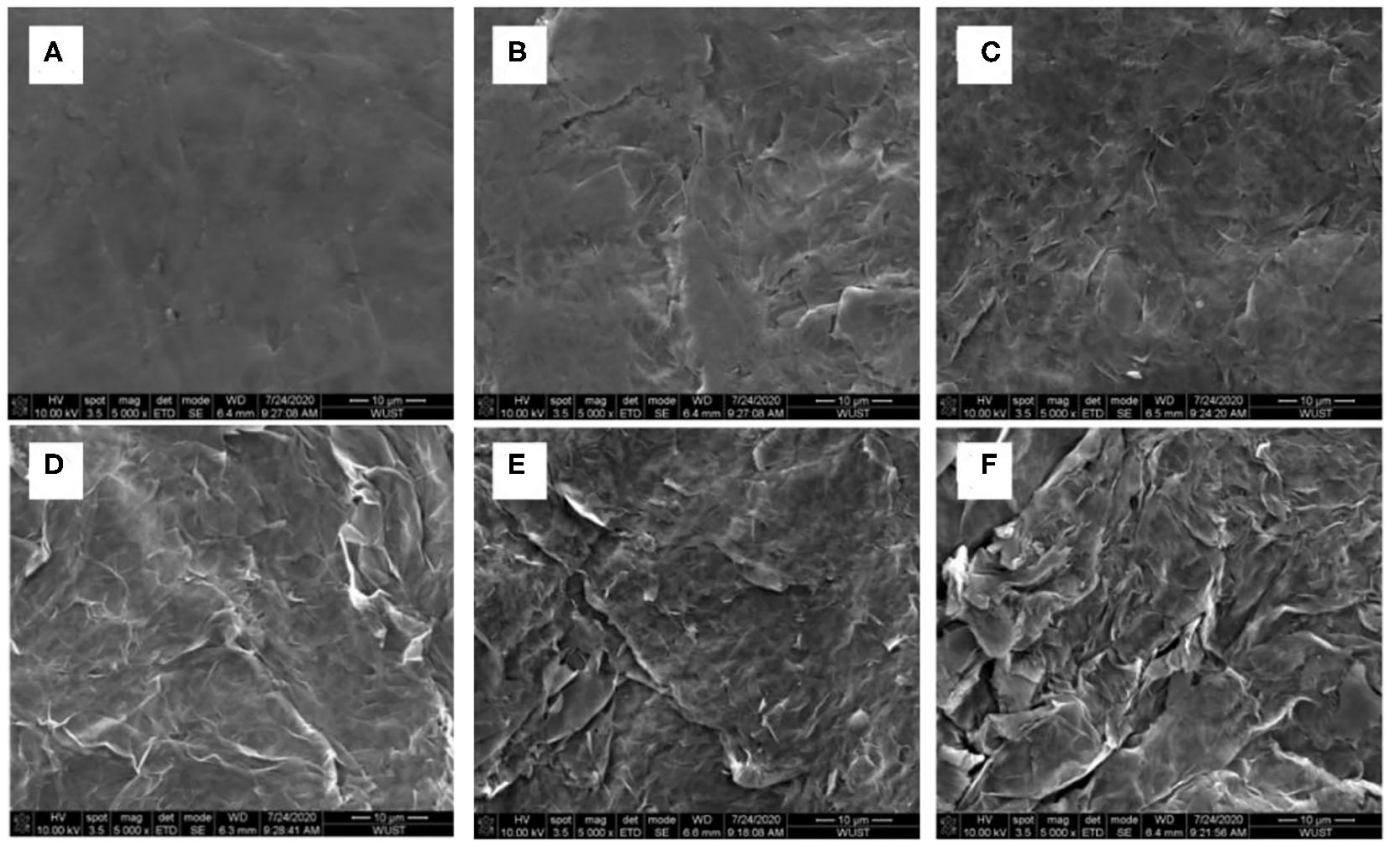
SEM images of **(A)** pristine graphite paper, **(B)** S1, **(C)** S2, **(D)** S3, **(E)** S4, and **(F)** S5.

Based on Figure 3B, realizing the poor performance by the non-activated surface of the graphite paper, electro-activation method has been adopted to activate the graphite paper surface. The respective treatment condition for samples of S1, S2, S3, S4, and S5 was stated in the experiment section. To visualize the effect of the activation result, SEM images have been collected and displayed in Figure 4. Through the comparison, from S1 to S5, there are more and more folds on the graphite paper surface as compared with pristine graphite paper in Figure 4A. The larger rugae area reflects an increased surface area. Hence, it is supposed to harvest enhanced ion storage capability when assembled in an ZIB cell. Meanwhile, we found that the treatment was sensitive to potential rather than the exposure time. As can be seen that the surface of S1 in Figure 4B is much smoother than S3 in Figure 4D. Even though, unlike S4 and S5, the surface of S3 still keeps as a whole with shallow wrinkles rather than fractures in Figures 4E,F. Accordingly, all samples show a certain degree of surface oxidization and sulfidation based on their EDS analysis. The overall content of oxygen is higher than sulfur (at. 12 vs. at. 2% on average) which was donated by ${\text{SO}}_{4}^{2-}$.

**Figure 5:**
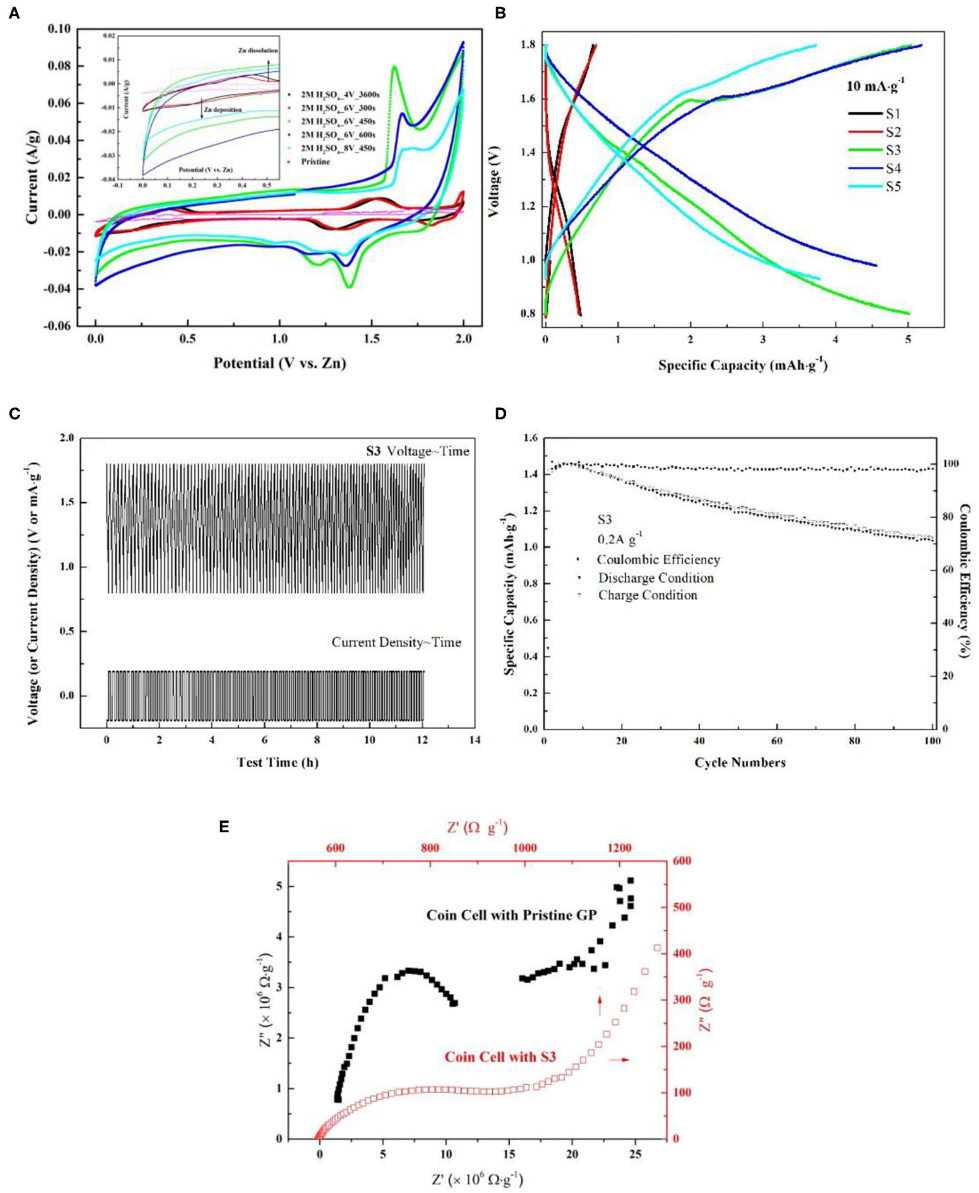
**(A)** Cyclic voltammetry comparison among pristine GP, S1, S2, S3, S4, and S5. The measurement was conducted by using CR2032 coin-type cell with Zn foil as counter electrode and 1 M ZnSO_4_ as electrolyte. The scan rate was 1 mV s^−1^; **(B)** Galvanostatic charge/discharge curves of AGP/Zn between 0.8 and 1.9 V at 10 mA g^−1^; **(C)** Gavanostatic cycling of S3 at 0.2 A g^−1^ for 100 cycles; **(D)** Cycling performance of the cell with S3 as cathode at 0.2 A g^−1^; **(E)** EIS Nyquist plots comparison between coin cell with pristine GP and S3. The data was captured under their respective open circuit potential.

To discuss the effects of activated graphite papers (AGP) on ZIB performance, CVs of the coin cell with non-activated (pristine GP) and different activated graphite paper (AGP) as cathode has been measured and compared. Figure 5A displays the CV curves of coin cells at 1 mV s^−1^. As compared to the CV of the cell with non-activated graphite paper (NAGP), the observed anodic peak in all cells with AGP migrates more negatively, which could be related to the electrode activation effects, similar to the observation of the initial profile changes in MnO_2_-based cathodes in ZIBs (Zhang et al., 2016). Further comparison finds that during the anodic sweeping, peaks centered at 1.55 and 1.50 V for S1 and S2, respectively, become sharp at 1.62, 1.67, and 1.7 V in CV of S3, S4, and S5 separately. The change is suspected to be induced by anions (${\text{SO}}_{4}^{2-}$) intercalation into graphite interlayers as discussed in zinc graphite battery (Fan et al., 2019). Moreover, through comparing the half peak width and anodic current density, the anodic peak located at 1.62 V shows the narrowest and highest, which implies a faster kinetic process on S3 interface than on the others'. In cathodic scan, a large cathodic peak centered at 1.29 and 1.34 V for S1 and S2, respectively, is converted into two distinctive peaks as captured in CV of S3, S4, and S5. Enlightened by the phenomenon observed in LiMn_2_O_4_, that two distinct cathodic peaks correspond to a two-step insertion of Li^+^ into a Li-rich and Li-depleted state (Lu et al., 2014; Suo et al., 2015), these two distinct cathodic peaks could be attributed to gradual insertion of Zn^2+^ into the near-surface and deep interlayer of AGP. Because that the specific area of all these three samples show great enhancement as compared with the other two and pristine one in Figure 4. Furthermore, as can be seen in the enlarged curves (Figure 5A, inset), the redox couple for deposition/dissolution of Zn^2+^/Zn is less significant due to the improved capacitive behavior after the activation. This benefit is given by the increased specific surface area as shown in Figure 4.

In order to assess ZIB performance with AGPs, galvanostatic charge/discharge curves of AGP/Zn between 0.8 and 1.9 V at 10 mA g^−1^ have been detected. From the comparison shown in Figure 5B, it points out that AGP of S3 shows the highest specific capacity of 5.0 mAh g^−1^ which is two orders of magnitude higher than the pristine one and one orders than S1 and S2. After the assessment, S3 is proved to be the optimized cathode in this study. Therefore, gavanostatic cycling of S3 at 0.2 A g^−1^ was conducted to evaluate its stability. As elaborated in Figure 5C, from the start to the end of 100 cycles, there is no changes on its voltage~ time profile which supports its good reversibility and high stability. Based on Figure 5C, coulombic efficiency of the cell with S3 has been calculated and was placed in Figure 5D. After 100 cycles of charge/discharge circulation, its coulombic efficiency can still attain at 96%. Hence, concluded from above discussion, GP activated under S3 condition gives good ZIB performance. To elucidate the extraordinary improvement of ZIB performance by using S3, EIS comparison in Figure 5E between coin cell with NAGP and S3 has been discussed. From the interfacial standpoint, it can be seen that the electrode resistance of coin cell with NAGP is four orders of magnitude higher than the one with S3. The high contact resistance should be due to the hydrophobic nature of graphite without the treatment. In addition, the large semi-circle diameter of coin cell with NAGP also presents a high charge transfer resistance. This observation is actually in agreement with the CV conclusion, the large half peak width and low current density of which tells the sluggish kinetic process in coin cell with NAGP. Due to this high charge transfer resistance, the coin cell with NAGP also shows low specific capacity as compared with that of S3. To conclude, from an interfacial perspective, the improvements of using S3 should be contributed to by the increased hydrophilic property of GP after the treatment, hence decreasing the contact resistance and corresponding charge transfer resistance. Finally, the coin cell with S3 exhibits an advantageous ZIB performance in this study.

**Figure 6:**
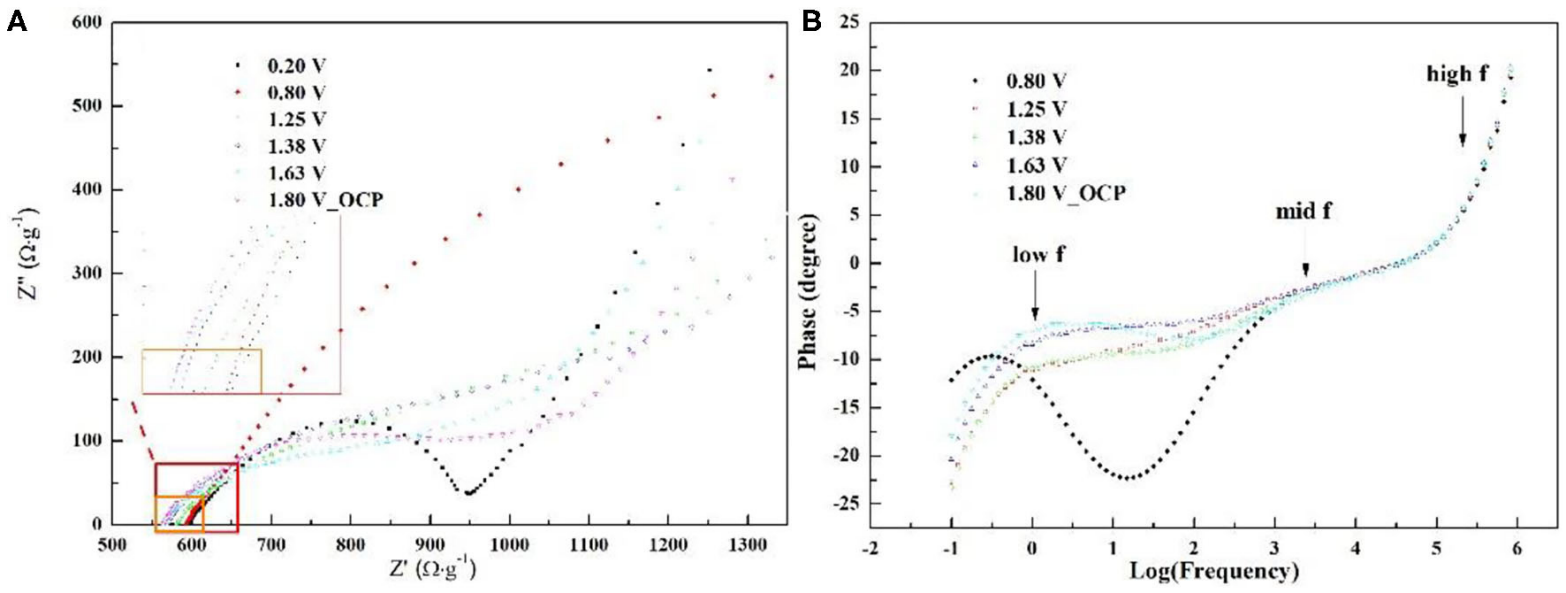
Nyquist plots **(A)** and Bode plots **(B)** of the ZIB with S3, Zn foil, and 1 M ZnSO_4_ in a CR2032 coin under different voltage (the chosen voltage was based on the platform and peak location in CV of S3).

To reveal the interfacial behavior of S3 in ZIB, the electrochemical impedance spectra of the coin cell under different voltage have been investigated and exhibited in Figure 6. The choice of voltage is based on the electrochemical process corresponding to CV of S3 in Figure 5A. The Nyquist plots in Figure 6A displays one depressed semi-circle which concatenates one semi-circle in mid frequency region, followed by a large semi-circle in low frequency at all voltages. The semi-circle in low frequency is related to peaks in the corresponding frequency region in Figure 6B. To quantitatively analyze the electrochemical interfacial process during ZIB operation via S3, Zn foil, and 1 M ZnSO_4_, the equivalent circuit in Figure 2C compatible with below impedance spectra is adopted and the corresponding fitting parameters are listed in Table 2. In the table, parameter of *Rs* represents the series resistance from electrode, solution, and contact; charge transfer resistance (*R*_*CT*_) with a parallel constant phase element (*CPE*) which relates to Zn^2+^/Zn reaction at the surface of the Zn foil along with extraction/insertion of Zn^2+^ from/into S3; the serial open circuit Warburg impedance (*Wo*) in the circuit reflects ion diffusion kinetics from electrolyte to the electrode/electrolyte interface. As shown, comparing parameters obtained at 1.25, 1.38, and 1.63 V, *R*_*S*_, *R*_*CT*_, and Warburg impedance *W*_*o*_*-R* shows the lowest value at 1.63 V. This decrease corresponds to the sharp peak in CV of S3 (Figure 5A), revealing the fast extraction of Zn^2+^ from S3 which possibly promotes the charge transfer rate for Zn^2+^ diffusion and Zn deposit. However, all these three values are high at 0.8 V. Combining with Figure 5A, a typical capacitive behavior is shown as the nature of carbon-based material. Therefore, the high resistance should originate from the double layer on the electrode surface. Consistently, a minimum *CPE-P* is obtained at 0.8 V due to the limited contribution from the double layer on near-surface. To conclude, during the operation window, the cell goes through a conversion from capacitive predominance to a fast charge transfer process involving extraction/insertion of Zn^2+^.

**Table 2:** List of EIS fitting parameters.

**Voltage**	***Rs***	***R_***CT***_***	***CPE-T***	***CPE-P***	***W_***O***_-R***	***W_***O***_-P***	***W_***O***_-T***
**V**	**Ω·g^**−1**^**	**Ω·g^**−1**^**	**F × g^**−1**^**		**Ω·g^**−1**^**	**s**	
0.8	592.6	2128.7	0.053	0.64	8.48	0.001	0.30
1.25	567.6	249.4	0.287	0.53	1377.78	4.917	0.34
1.38	556.5	305.4	0.160	0.56	1359.57	4.413	0.36
1.63	553.7	221.7	0.185	0.54	834.57	3.762	0.35

## Conclusion

The interfacial effect of three kinds of inorganic mild electrolyte on ZIBs performance have been investigated by electrochemical techniques. The construction of ZIBs includes Zn foil, graphite paper, and the chosen electrolyte. Through the comparison, it reveals that ZnSO_4_ is the most preferred electrolyte among the chosen three. It provides faster Zn stripping/plating kinetics. And the electrolyte of Zn(CH_3_COO)_2_ exhibited the most promising alternative in the ZIB system. However, ZIB with Zn(NO_3_)_2_ as electrolyte exhibits poor performance. Even though the overall ZIB performance based on pristine graphite paper presents limited specific capacity. Therefore, an electro-activation method has been adopted for the activation of graphite paper. After the optimization, through the comparison, it was found that sample S3, activated at 6 V for 450 s in 2 M H_2_SO_4_, displays outstanding improvements. After the activation, firstly, the surface of S3 becomes rougher than the pristine one but intact as a whole rather than exhibiting the breaks seen in other samples; secondly, the coherency of its surface ensures a fast charge transfer kinetics reflected by CV with high current density and narrow half peak width; thirdly, the specific capacity based on CR2032 coin with S3 exhibits two orders of magnitude higher than the one with pristine graphite paper. Moreover, coin cell CR2032 with S3 also shows the highest specific capacity in this study.

## Data Availability Statement

All datasets generated for this study are included in the article/supplementary material.

## Author Contributions

YG designed experiments, carried out experiments, analyzed experimental results, and wrote the manuscript. BF supported required experimental instruments and helped revising the manuscript. YL supported required experimental instruments, publish payment, and helped revising the final manuscript. All authors contributed to the article and approved the submitted version.

## Conflict of Interest

The authors declare that the research was conducted in the absence of any commercial or financial relationships that could be construed as a potential conflict of interest.

## References

[B1] Armand M. Tarascon J. M. (2008). Building better batteries. Nature 451, 652–657. 10.1038/451652a 18256660

[B2] Cheng F. Chen J. Gou X. Shen P. (2005). High-power alkaline Zn-MnO_2_ batteries using γ-MnO_2_ nanowires/nanotubes and electrolytic zinc powder. Adv. Mater. 17, 2753–2756. 10.1002/adma.200500663

[B3] Choi J. W. Aurbach D. (2016). Promise and reality of post-lithium-ion batteries with high energy densities. Nat. Rev. Mater. 1:16013 10.1038/natrevmats.2016.13

[B4] Fan J. X. Xiao Q. Q. Fang Y. B. Li L. Yuan W. H. (2019) A rechargeable zn/graphite dual-ion battery with an ionic liquid-based electrolyte. Ionics 25, 1303–1313. 10.1007/s11581-018-2644-x

[B5] Gui Y. Blackwood D. J. (2015). Honey-comb structured WO_3_/TiO_2_ thin films with improved. J. Electrochem. Soc. 162, E205–E212. 10.1149/2.0031510jes

[B6] Hwang J. (2018). Symmetric cell electrochemical impedance spectroscopy of Na_2_FeP_2_O_7_ positive electrode material in ionic liquid electrolytes. J. Phys. Chem. C 122, 26857–26864. 10.1021/acs.jpcc.8b09233

[B7] Kim H. Hong J. Park K. Y. Kim H. Kim S. W. Kang K. (2014). Aqueous rechargeable li and na ion batteries. Chem. Rev. 114, 11788–11827. 10.1021/cr500232y 25211308

[B8] Konarov A. K. A. Voronina N. Jo J. H. Bakenov Z. Sun Y. K. Myung S. T. (2018). Present and future perspective on electrode materials for rechargeable zinc-ion batteries, ACS Energy Lett. 3, 2620–2640. 10.1021/acsenergylett.8b01552

[B9] Lee B. Lee H. R. Kim H. Chung K. Y. Cho B. W. Oh S. H. (2015). Elucidating the intercalation mechanism of zinc Ions into α-MnO_2_ for rechargeable zinc batteries. Chem. Commun. 51, 9265–9268. 10.1039/C5CC02585K 25920416

[B10] Lee B. Seo H. R. Lee H. R. Yoon C. S. Kim J. H. Chung K. Y.. (2016). Critical role of ph evolution of electrolyte in the reaction mechanism for rechargeable zinc batteries. Chem. Sus. Chem. 9, 2948–2956. 10.1002/cssc.201600702 27650037

[B11] Li G. Yang Z. Jiang Y. Jin C. Huang W. Ding X. (2016). Towards polyvalent ion batteries: a zinc-ion battery based on NASICON structured Na_3_V_2_(PO_4_)_3_. Nano Energy 25, 211–217. 10.1016/j.nanoen.2016.04.051

[B12] Li S. Dong Y. Xu L. Xu X. He L. Mai L. (2014). Effect of carbon matrix dimensions on the electrochemical properties of Na_3_V_2_(PO_4_)_3_ nanograins for high-performance symmetric sodium-ion batteries. Adv. Mater. 26, 3545–3553. 10.1002/adma.201305522 24633680

[B13] Lin M. C. Gong M. Lu B. Wu Y. Wang D. Y. Guan M.. (2015). An ultrafast rechargeable aluminium-ion battery. Nature 520, 324–328. 10.1038/nature14340 25849777

[B14] Liu Z. Bertram P. Endres F. (2017). Bio-degradable zinc-ion battery based on a prussian blue analogue cathode and a bio-ionic liquid-based electrolyte. J. Solid State Electrochem. 21, 2021–2027. 10.1007/s10008-017-3589-0

[B15] Liu Z. Pulletikurthi G. Endres F. (2016). A prussian blue/zinc secondary battery with a bio-ionic liquid-water mixture as electrolyte. ACS Appl. Mater. Interfaces 8, 12158–12164. 10.1021/acsami.6b01592 27119430

[B16] Lu J. Zhan C. Wu T. Wen J. Lei Y. Kropf A. J.. (2014). Effectively suppressing dissolution of manganese from spinel lithium manganate via a nanoscale surface-doping approach. Nat. Commun. 5:5693. 10.1038/ncomms6693 25514346

[B17] Lu Y. Goodenough J. B. Kim Y. (2011). Aqueous cathode for next-generation alkali-ion batteries. J. Am. Chem. Soc. 133, 5756–5769. 10.1021/ja201118f 21443190

[B18] Muldoon J. Bucur C. Gregory T. (2014). Quest for nonaqueous multivalent secondary batteries: magnesium and beyond. Chem. Rev. 114, 11683–11720. 10.1021/cr500049y 25343313

[B19] Pan H. Shao Y. Yan P. Cheng Y. Han K. S. Nie Z. (2016). Reversible aqueous zinc/manganese oxide energy storage from conversion reactions. Nat. Energy 1:16039 10.1038/nenergy.2016.39

[B20] Parker J. F. Chervin C. N. Pala I. R. Machler M. Burz M. F. Long J. W.. (2017). Rechargeable nickel-3D zinc batteries: an energy-dense, safer alternative to lithium-ion Science 356, 415–418. 10.1126/science.aak9991 28450638

[B21] Ponrouch A. Frontera C. Barde F. Palacin M. R. (2016). Towards a calcium-based rechargeable battery. Nat. Mater. 15, 169–172. 10.1038/nmat4462 26501412

[B22] Qiu H. Y. Du X. F. Zhao J. W. Wang Y. T. Ju J. W. Chen Z.. (2019). Zinc anode-compatible in-situ solid electrolyte interphase via cation solvation modulation. *Nat*. Comm. 10:5374. 10.1038/s41467-019-13436-3 31772177 PMC6879498

[B23] Suo L. Borodin O. Gao T. Olguin M. Ho J. Fan X.. (2015). “Water-in-Salt” electrolyte enables high-voltage aqueous lithium-ion chemistries. Science 350, 938–943. 10.1126/science.aab1595 26586759

[B24] Wang C. Appleby A. J. Little F. E. (2001). Electrochemical study on nano-Sn, Li_4.4_ Sn and AlSi_0.1_ powders used as secondary lithium battery anodes. J. Power Sources 93, 174–185. 10.1016/S0378-7753(00)00576-0

[B25] Wang F. Borodin O. Gao T. Fan X. L. Sun W. Han F. D.. (2018). Highly reversible zinc metal anode for aqueous batteries. Nat. Mat. 17, 543–549. 10.1038/s41563-018-0063-z 29662160

[B26] Wang Z. Li H. Tang Z. Liu Z. Ruan Z. Ma L. (2018). Hydrogel electrolytes for flexible aqueous energy storage devices. Adv. Funct. Mater. 28:1804560 10.1002/adfm.201804560

[B27] Whittingham M. S. (2004). Lithium batteries and cathode materials. Chem. Rev. 104, 4271–4302. 10.1021/cr020731c 15669156

[B28] Xu C. Li B. Du H. Kang F. Y. (2012). Energetic zinc ion chemistry: the rechargeable zinc ion battery. Angew. Chem. Int. Ed. Engl. 51, 933–935. 10.1002/anie.201106307 22170816

[B29] Yabuuchi N. Kubota K. Dahbi M. Komaba S. (2014). Research development on sodium-ion battery. Chem. Rev. 114, 11636–11682. 10.1021/cr500192f 25390643

[B30] Yamamoto T. Shoji T. (1986). Rechargeable Zn|ZnSO_4_|MnO_2_-type cells. Inorg. Chim. Acta 117, L27–L28. 10.1016/S0020-1693(00)82175-1

[B31] Yang C. Lin S. (2002). Improvement of high-rate capability of alkaline Zn-MnO_2_ battery. J. Power Sources 112, 174–183. 10.1016/S0378-7753(02)00354-3

[B32] Yuan D. Manalastas W. Jr„ Zhang, L. P. Chan J. J. Meng S. Z. Chen Y. Q.. (2019). Lignin@ nafion membranes forming zn solid-electrolyte interfaces enhance the cycle life for rechargeable zinc-ion batteries. ChemSusChem 12, 4889–4900. 10.1002/cssc.201901409 31475452

[B33] Zhang N. Cheng F. Y. Liu Y. C. Zhao Q. Lei K. X. Chen C. C.. (2016). Cation-deficient spinel ZnMn_2_O_4_ cathode in Zn(CF_3_SO_3_)_2_ electrolyte for rechargeable aqueous zn-ion battery. J. Am. Chem. Soc. 138, 12894–12901. 10.1021/jacs.6b05958 27627103

[B34] Zhao H. Xu J. Yin D. Du Y. (2018). Electrolytes for batteries with earth-abundant metal anodes. Chem. Eur. J. 24, 18220–18234. 10.1002/chem.201802438 30044015

[B35] Zhao J. Ren H. Liang Q. H. Yang D. Xi S. B. Wu C. (2019). High-performance flexible quasi-solid-state zinc-ion batteries with layer-expanded vanadium oxide cathode and zinc/stainless steel mesh composite anode. Nano Energy 62, 94–102. 10.1016/j.nanoen.2019.05.010

